# Pharmacokinetics of a continuous intravenous infusion of hydromorphone in healthy dogs

**DOI:** 10.3389/fvets.2024.1362730

**Published:** 2024-04-15

**Authors:** Candace Wimbish, Alex M. Lynch, Heather K. Knych, Yu Ueda, Kristen M. Messenger

**Affiliations:** ^1^Department of Clinical Sciences, College of Veterinary Medicine, NC State University, Raleigh, NC, United States; ^2^K.L. Maddy Equine Analytical Chemistry Laboratory, School of Veterinary Medicine, University of California, Davis, Davis, CA, United States; ^3^Department of Molecular Biomedical Sciences, College of Veterinary Medicine, NC State University, Raleigh, NC, United States

**Keywords:** constant rate infusion, hydromorphone, dog, pharmacokinetics, pain, opioid

## Abstract

**Introduction:**

Dosing recommendations for hydromorphone intravenous constant rate infusion (IV CRI) are derived from simulations following IV bolus administration. While this extrapolated dose regimen has been described clinically, pharmacokinetics (PK) of hydromorphone infusions in dogs are not yet described. The study objective was to describe the PK of hydromorphone in healthy dogs receiving an IV bolus followed by an IV CRI for 48 h.

**Methods:**

A prospective, experimental study was performed involving the administration of hydromorphone (0.1 mg/kg IV bolus then IV CRI 0.01 mg/kg/h over a 48 h period) to 6 healthy Beagle dogs. Blood samples were collected at 16 time points between 0 and 58 h relative to the initial bolus. Plasma hydromorphone concentrations were analyzed by high pressure liquid chromatography with tandem mass spectrometry detection. Pharmacokinetic parameter estimates were obtained with compartmental methods using commercially available software.

**Results:**

A two-compartment model with first order elimination was used. At the end of the infusion, median (range) plasma hydromorphone concentrations were 6.8 (5.5–19.6) ng/mL. The median total body clearance was 30.4 (19.8–36.7) mL/min/kg; volume of distribution at steady state was 4.5 (3.2–7.8) L/kg; and terminal elimination half-life was 11.2 (7.6–24.3) h.

**Conclusion:**

Hydromorphone (0.1 mg/kg IV bolus then IV CRI of 0.01 mg/kg/h) maintained steady-state plasma concentrations above the minimum human analgesic target in healthy Beagle dogs with minimal side effects. Further studies are needed to determine the effective plasma concentrations of hydromorphone in painful dogs.

## 1 Introduction

Unmitigated pain can result in adverse physiologic effects that increase patient morbidity, as well as negatively impact welfare (1, 2). Opioids are at the core of effective acute pain management strategies in dogs, with hydromorphone being used by 72% of veterinary practitioners for this purpose (3). Hydromorphone has a large volume of distribution and is rapidly cleared in dogs, meaning frequent bolus dosing is necessary to achieve sustained therapeutically effective plasma concentrations (4). Hydromorphone administered at a dose of 0.1 mg/kg IV every 2 h provides an adequate antinociceptive effect in dogs (4–6). While intravenous (IV) bolus administration of hydromorphone is cost-effective and reliable, repeated bolus administration is labor intensive, risks peaks and troughs in analgesic effect, and is associated with adverse effects including nausea, emesis, vocalization, and panting (7). An IV continuous rate infusion (CRI) of hydromorphone could overcome some, if not all, of these issues. There is also an emerging need to develop novel opioid dosing regimens for small animals. Some opioids are prohibitively expensive in large dogs, or there have been recent supply chain issues leading to inconsistent opioid availability, and lastly, there are serious concerns over the use of certain opioids due to risk of human diversion (3).

The pharmacokinetics (PK) of several opioid IV CRI protocols have been reported in dogs, but the PK of hydromorphone administered by this route is yet to be investigated, despite clinical descriptions of its use in dogs (7). Previous PK studies suggest IV CRI rates of 0.03 mg/kg/h to target and maintain plasma concentrations of 4 ng/mL, but these recommendations are based on single IV bolus dosing studies where clearance was found to be very high following a single dose (5, 6). These suggested rates might be too high based on pilot work performed by our group in a study evaluating single IV bolus of hydromorphone and using a continuous subcutaneous administration device (8). Fentanyl infusions have variable accumulation in dogs depending on the duration of infusion, a phenomenon described as the *context-sensitive half-time*, such that the longer the infusion, the longer the elimination half-life and thus time to offset of effect (9). It is plausible, but unknown, if hydromorphone administered by CRI could behave similarly.

The primary goal of this study, therefore, was to describe the PK parameters of hydromorphone following a 48-h IV CRI administered to healthy dogs. The secondary aims of the study were to evaluate serial nausea and sedation scores in the dogs administered hydromorphone.

## 2 Materials and methods

### 2.1 Animals

Six purpose-bred Beagle dogs were enrolled. There were 4 male neutered and 2 female spayed dogs, with a mean weight of 12.2 ± 2.6 kg and a mean age of 5.8 ± 2.6 years. These dogs were assessed as healthy based on physical examination findings and routine laboratory analyses, including complete blood count and serum biochemistry profiles. The dogs were housed in the NC State University Laboratory Animal Resources Facility, where a maintenance diet was provided twice daily, along with water *ad libitum*. The study was approved by the Institutional Animal Care and Use Committee (Protocol number 19-814-O).

### 2.2 Study design

A prospective experimental design was used for the study. One day prior to the start of the study, all dogs were sedated with 125 mg/m^2^ dexmedetomidine^1^ administered by intramuscular injection to facilitate the placement of a 18-Ga, 12-cm single lumen catheter for serial blood sampling^2^ into the right or left external jugular vein using a modified Seldinger technique (10). After successful placement of the sampling catheter, each dog received 0.05 mg/kg of atipamezole by intramuscular injection^3^ to antagonize the effects of dexmedetomidine. The sampling catheters were maintained by flushing with 0.9% saline at 6-h intervals. On the day of study commencement, an 18-Ga peripheral IV catheter^4^ was placed into either the right or left cephalic vein of each dog to be used for hydromorphone^5^ administration.

Each dog received a bolus of hydromorphone 0.1 mg/kg IV, immediately followed by an IV CRI at a rate of 0.01 mg/kg/h for a continuous 48-h period. The dose of 0.01 mg/kg/h was based on preliminary work performed by our group (8). Blood samples were obtained from the pre-placed jugular catheters at 12 time points relative to hydromorphone bolus administration, corresponding to baseline (0 h), 0.08 h, 1 h, 2 h, 4 h, 8 h, 12 h, 18 h, 24 h, 30 h, 32 h, 40 h and 48 h. At the 48-h time mark, the hydromorphone IV CRI was discontinued, and a further 4 blood samples were obtained during the subsequent 10 h. These corresponded to 50 h, 52 h, 55 h and 58 h relative to the initial hydromorphone bolus. The sampling catheters were removed after collecting the final blood sample.

The dogs were monitored continuously over the study period. At each time point, vital signs (heart and respiratory rates) were recorded, along with previously described sedation and nausea scores in dogs (11, 12). Briefly, the sedation score consisted of serial evaluation of seven patient characteristics: spontaneous posture; palpebral reflex; eye position; jaw and tongue relaxation; response to auditory stimulation; resistance to being laid in lateral recumbency; and overall demeanor (Supplementary Table S1). The nausea score, which was always performed prior to sedation scoring, involved a visual analog scale based upon operator evaluation of signs of salivation, lip licking, lethargy, restlessness, circling behavior, and vomiting (Supplementary Table S2).

In addition, any issues related to drug administration (e.g., obstructions to IV CRI flow) were recorded if they occurred.

### 2.3 Blood sampling and PK analysis

Blood samples collected at each time point were transferred into plastic tubes containing lithium heparin^6^ and placed on ice. Samples were centrifuged at 3,500 ×*g* for 10 min at 4°C within 60-min of collection. The plasma from centrifuged samples was separated and transferred to storage cryovials^7^ and stored at −80°C until analysis within 2 months of collection.

Hydromorphone concentrations were measured using previously published liquid chromatography tandem mass spectrometry (LC–MS/MS) methods (13). Briefly, for hydromorphone quantification, a partial validation was performed using canine plasma as the matrix. For both analytes, calibrators and negative control samples were prepared fresh for each quantitative assay. In addition, quality control samples [drug-free canine plasma fortified with hydromorphone at three concentrations within the standard curve, high, medium and low (160, 35, 0.3 ng/mL, respectively) were included with each sample set as an additional check of accuracy]. The limit of quantification (LOQ) was 0.1 ng/mL. The limit of detection (LOD) was 0.05 ng/mL. D3-hydromorphone was used as an internal standard. The response for hydromorphone was linear with a correlation coefficient of 0.99 between 0.1–200 ng/mL, and all calibrators and quality controls back-calculated to within ±15% of the nominal concentrations. Pharmacokinetic parameters for hydromorphone were obtained using commercially available software.^8^ Different compartmental models and weighting options were assessed for goodness of fit by visual inspection of the observed versus predicted plots, weighted residual versus observed and predicted plots, and comparison of Akaike Information Criterion values. All data were included in the pharmacokinetic models, including known outlier samples when infusion lines were found to be occluded. A two compartment IV bolus IV infusion model parameterized by microrate constants and weighted by 1/Y*Y was determined to best describe the data.

### 2.4 Statistical analysis

Descriptive statistics were used to summarize the plasma concentrations of hydromorphone and the pharmacokinetic results.^9^ A target hydromorphone plasma concentration of 4 ng/mL was used to indicate minimum concentrations for efficacy, based upon human data indicating an antinociceptive effect at this concentration, although no pharmacodynamic studies have confirmed this target (14, 15).

## 3 Results

**Figure 1:**
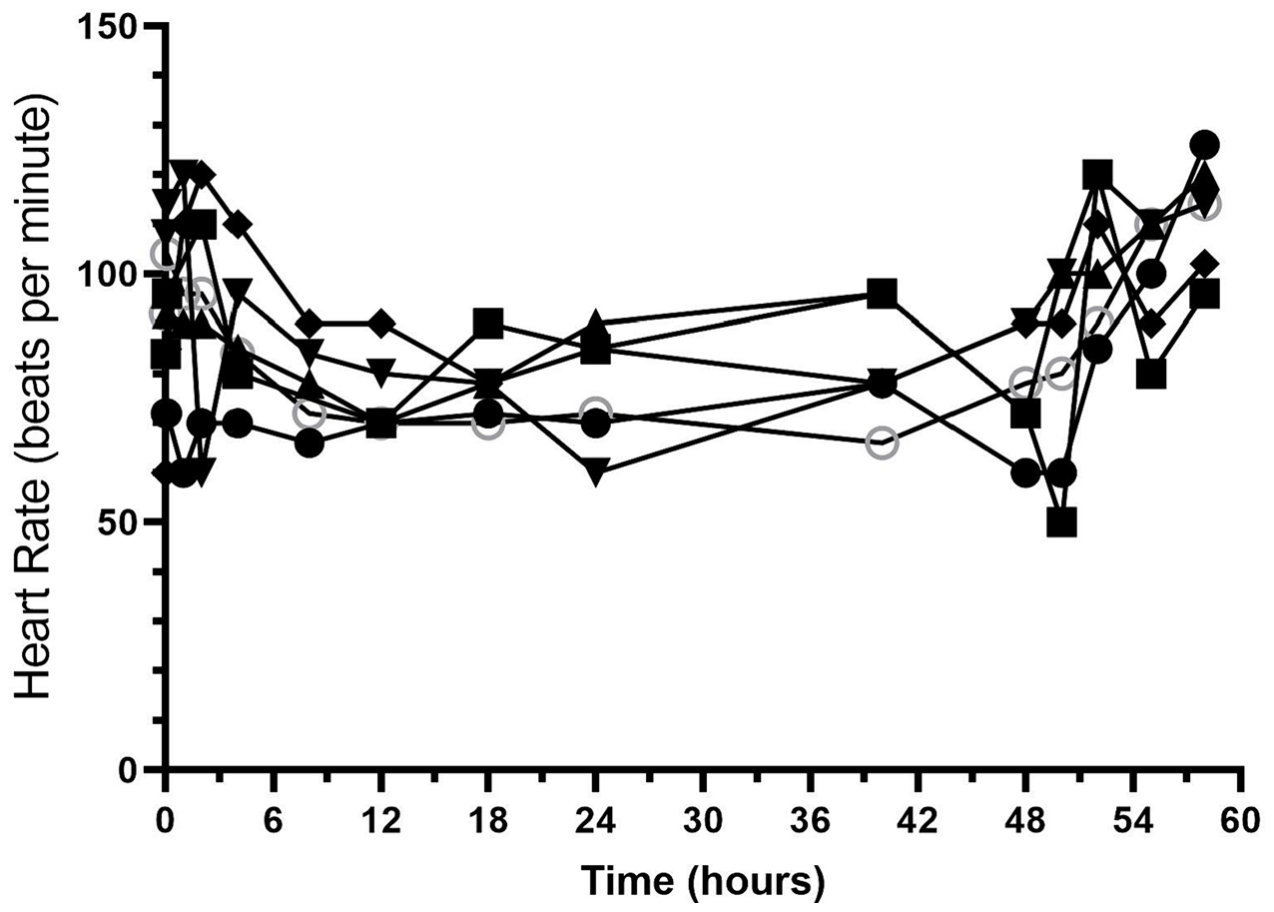
Heart rate (beats/min) versus time (hours) for 6 dogs over the duration of the study (0–60 h). Each dog was administered an intravenous bolus of hydromorphone (0.1 mg/kg) followed by a hydromorphone intravenous constant rate infusion (0.01 mg/kg/h) for 48-h.

**Figure 2:**
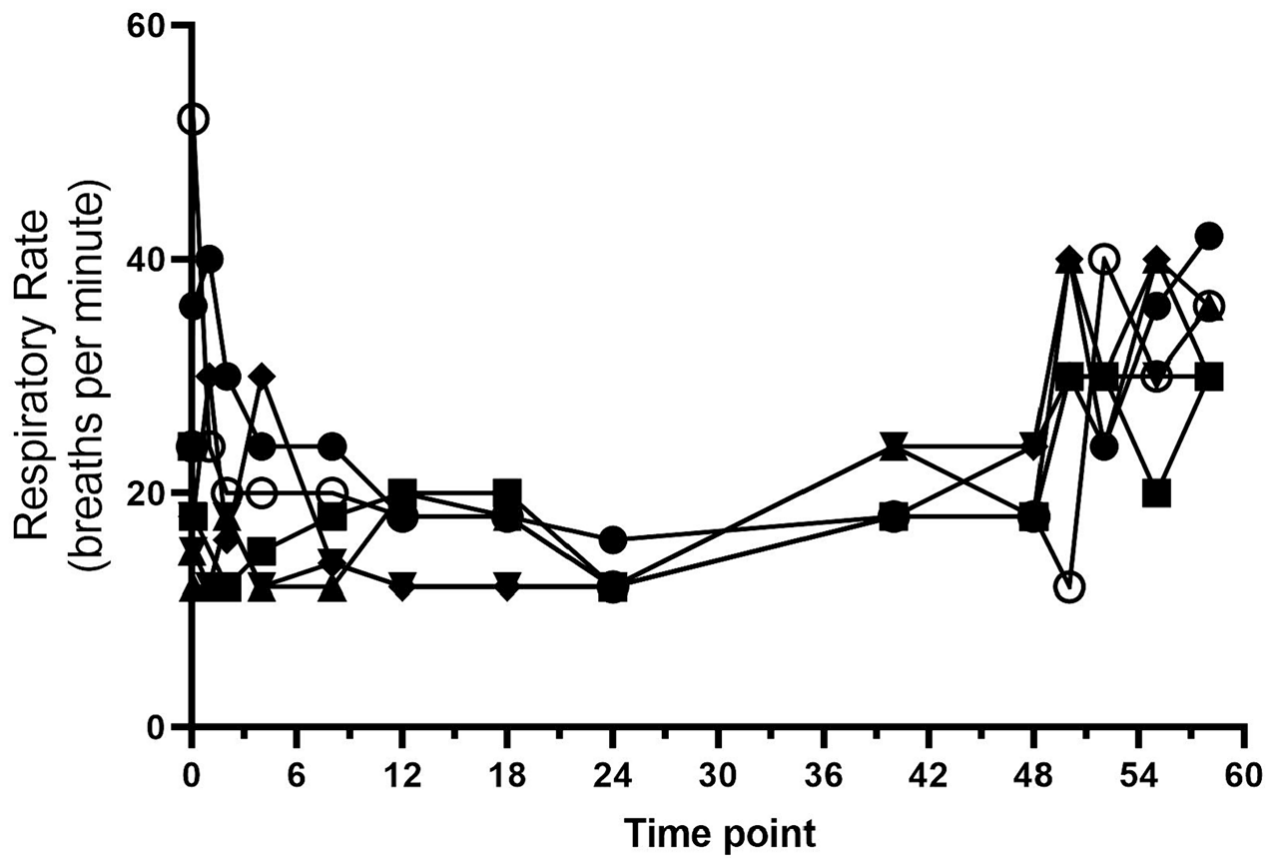
Respiratory rate (breaths/min) versus time (hours) for 6 dogs over the duration of the study (0–60 h). Each dog was administered an intravenous bolus of hydromorphone (0.1 mg/kg) followed by a hydromorphone intravenous constant rate infusion (0.01 mg/kg/h) for 48-h.

All dogs completed the study without any serious adverse effects. Heart rate and respiratory rate over the course of the study are shown in Figures 1, 2. The median (range) heart rate during hydromorphone administration was 88 (50–126) beats/min, while the median (range) respiratory rate was 19 (12–52) breaths/min. Panting was noted after initial hydromorphone bolus in each dog but subsided without further panting episodes during the remaining study period.

**Figure 3:**
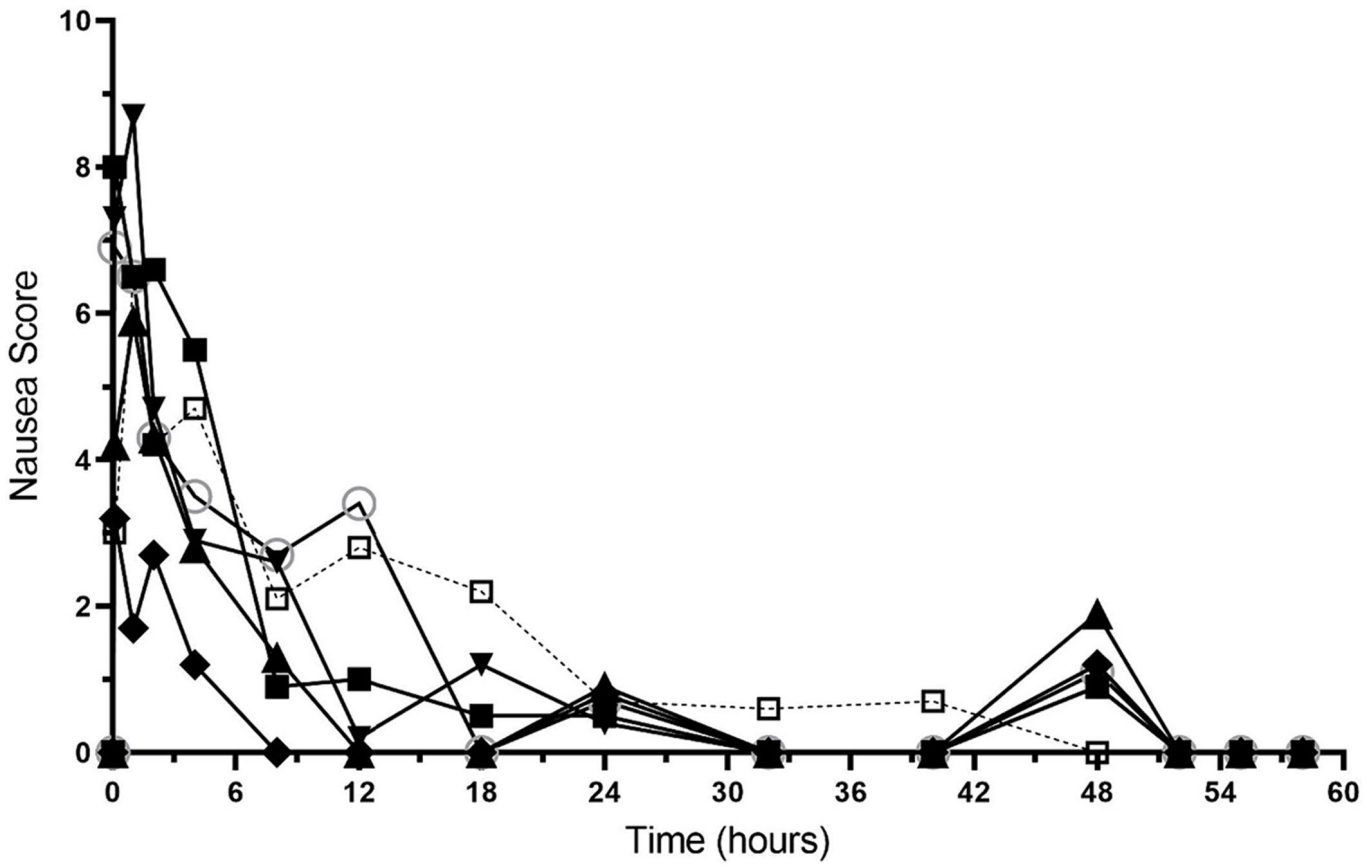
Nausea scores versus time (hours) for 6 dogs over the duration of the study (0–60 h). Each dog was administered an intravenous bolus of hydromorphone (0.1 mg/kg) followed by a hydromorphone intravenous constant rate infusion (0.01 mg/kg/h) for 48-h.

Sedation scores are shown in Figure 3 and Supplementary Table S2. The median (range) sedation score at baseline was 1 (1–2). Sedation scores increased after hydromorphone administration and remained higher than the baseline values for the duration of the hydromorphone CRI administration. The median (range) sedation score during the hydromorphone CRI was 6 (2.5–11). The median (range) sedation score after hydromorphone discontinuation was 0.8 (0–6).

**Figure 4:**
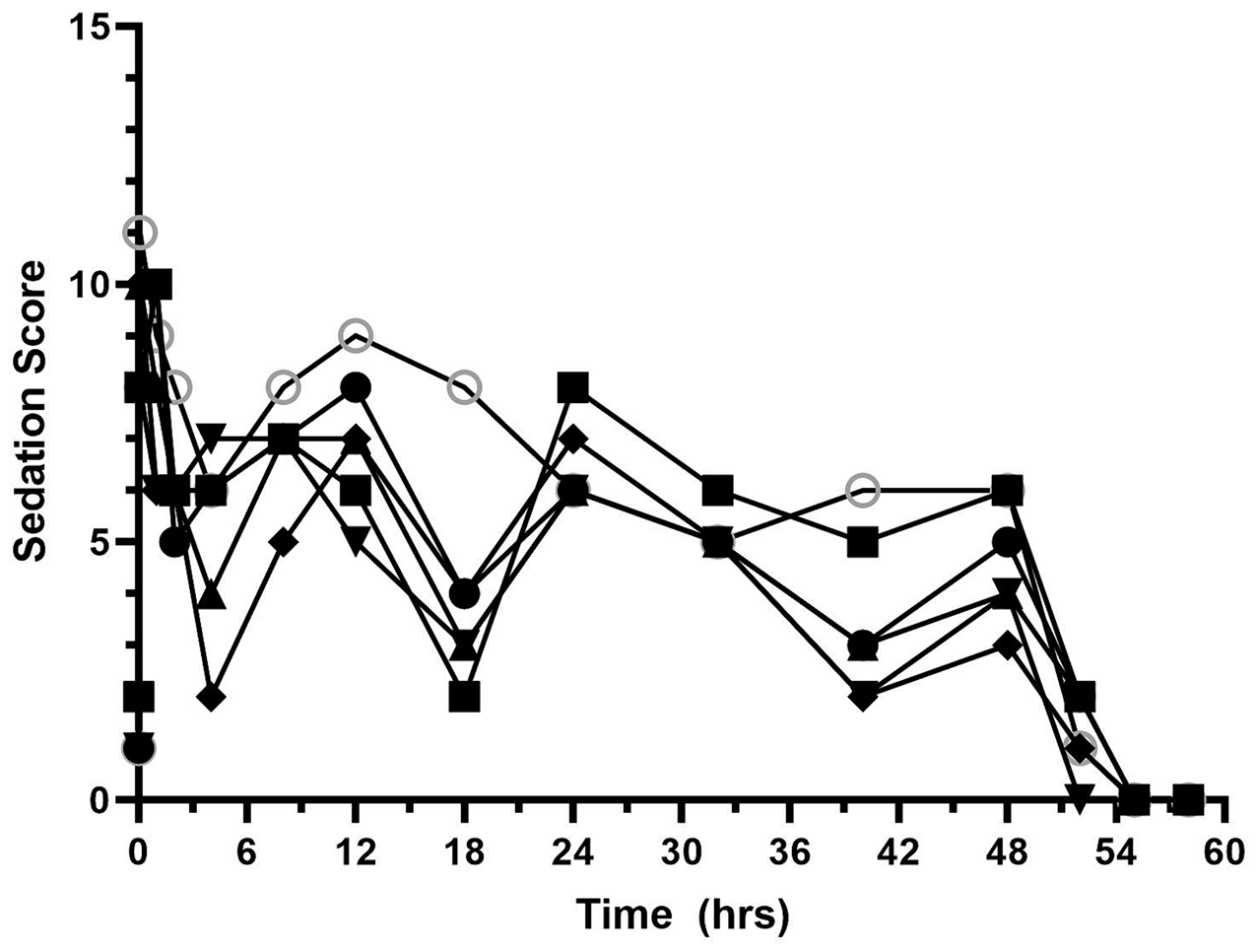
Sedation scores versus time (hours) for 6 dogs over the duration of the study (0–60 h). Each dog was administered an intravenous bolus of hydromorphone (0.1 mg/kg) followed by a hydromorphone intravenous constant rate infusion (0.01 mg/kg/h) for 48-h.

Nausea scores are shown in Figure 4 and Supplementary Table S3. All dogs had nausea scores of 0 (no nausea) at baseline, which increased after hydromorphone bolus administration. The median (range) nausea scores were 4.9 (1.2–8.7) for the first 4 h after hydromorphone administration and 0.5 (0–3.5) between the 8–48 h timepoints. Nausea scores of 0 were recorded after hydromorphone infusion discontinuation.

### 3.1 Pharmacokinetics

**Figure 5:**
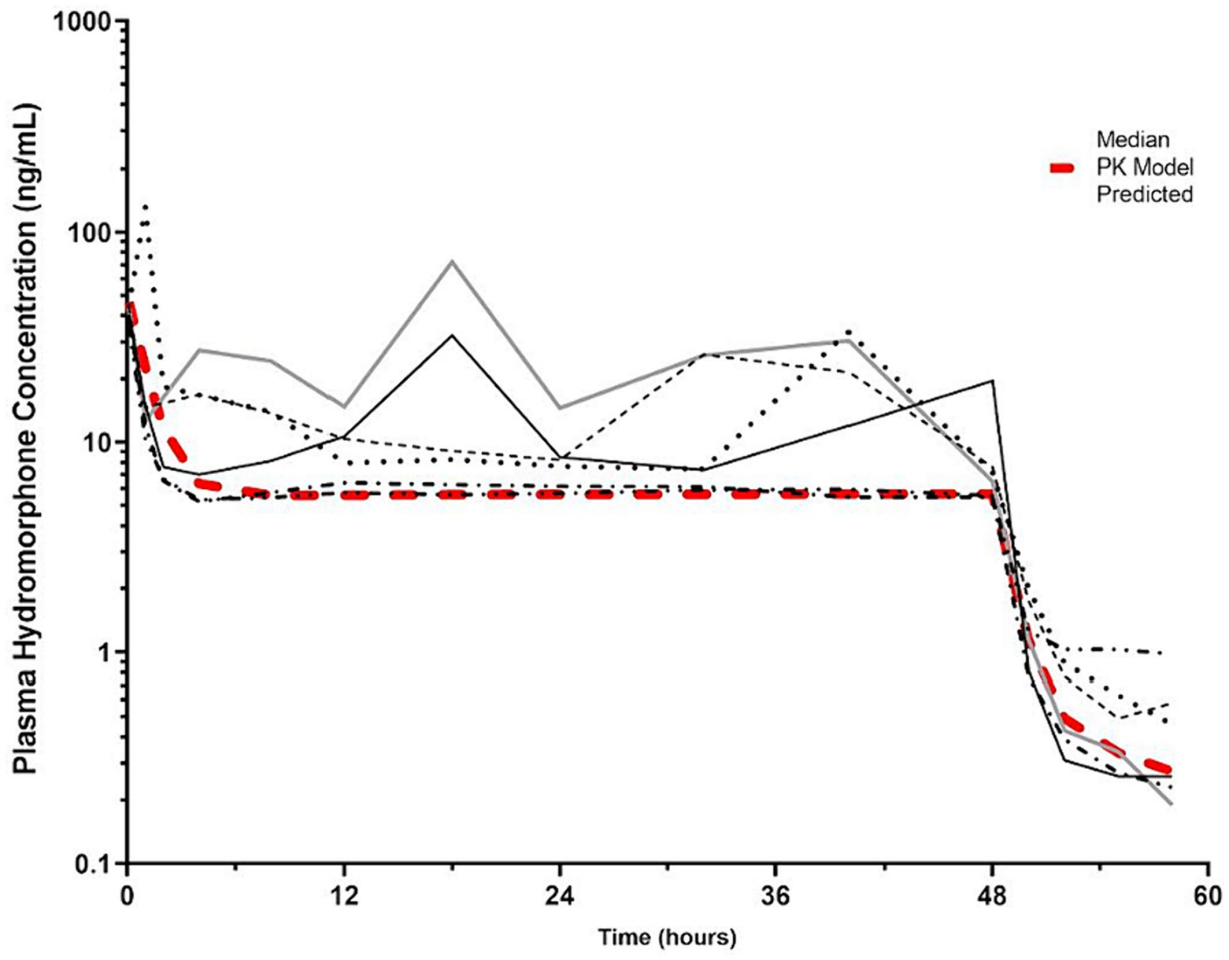
Hydromorphone plasma concentrations (ng/mL) versus time (hours) for 6 dogs over the duration of the study (0–60 h). Each dog was administered an intravenous bolus of hydromorphone (0.1 mg/kg) followed by a hydromorphone intravenous constant rate infusion (0.01 mg/kg/h) for 48-h. The pharmacokinetic predicted model is indicated by the red hashed line. The plasma concentration (4 ng/mL) reported to have the minimal antinociceptive effect in people is indicated by the dotted line (15).

By the end of the IV CRI, median plasma hydromorphone concentrations were 6.8 ng/mL (min-max 5.5–19.6 ng/mL). There was some variability in plasma drug concentrations over time in some dogs (Figure 5; Supplementary Figure S1), although occasional increases in concentrations during the infusions were noted in some dogs after correcting infusion line obstructions. An IV CRI administered at 0.01 mg/kg/h resulted in plasma hydromorphone concentrations above the human therapeutic target (4 mg/mL) for the duration of the study. Hydromorphone concentrations rapidly decreased after discontinuation. Pharmacokinetic parameters derived from the data are shown in Table 1.

**Table 1:** Pharmacokinetic parameters following 0.1 mg/kg intravenous bolus of hydromorphone followed by an intravenous constant rate infusion of 0.01 mg/kg/h of hydromorphone for 48-h in 6 dogs.

Parameter	Units	Median (range)
A	ng/mL	49.6 (49.3–58.0)
α	1/h	1.05 (0.73–1.24)
B	ng/mL	0.4 (0.2–0.7)
β	1/h	0.06 (0.03–0.10)
Elimination rate constant (K10)	1/h	0.9 (0.7–1.1)
Rate constant from central to peripheral compartment (K12)	1/h	0.08 (0.06–0.15)
Rate constant from peripheral to central compartment (K21)	1/h	0.07 (0.03–0.10)
Clearance (Cl)	mL/min/kg	30.4 (19.8–36.7)
Intercompartment Cl	mL/min/kg	2.7 (1.8–5.0)
Volume of the central compartment (Vc)	L/kg	2.0 (1.7–2.0)
Volume of distribution at steady state (Vd_ss_)	L/kg	4.5 (3.2–7.8)
Elimination half life (T1/2_k10_)	h	0.8 (0.6–1.0)
Distribution half life (T1/2_α_)	h	0.7 (0.6–1.0)
Terminal elimination half life (T1/2_β_)	h	11.2 (7.2–24.3)

## 4 Discussion

Our prospective experimental study was designed to characterize the PK profiles of hydromorphone administered by IV CRI over a 48-h period in healthy dogs. Our data identified that an initial IV bolus of 0.1 mg/kg hydromorphone, immediately followed by an IV CRI of 0.01 mg/kg/h, maintained steady state plasma concentrations between 8 and 12 ng/mL. This is above the minimum effective plasma concentration associated with an antinociceptive effect in people (4 ng/mL) (14, 15). Minimal adverse effects were seen in the dogs administered the hydromorphone IV CRI in this study. Dogs were mildly sedated but with minimal impact on respiratory rate. A short duration of nausea was observed following hydromorphone administration. There was no apparent drug accumulation during the infusion and plasma concentrations decreased quickly once the infusion was discontinued. While the terminal elimination half-life (T1/2_β_) was calculated to be nearly 12 h, this finding is likely due to the sensitivity of the assay used in this study (LOQ of 0.1 ng/mL compared with higher concentrations in older reports) and these plasma concentrations are unlikely to provide any clinically relevant analgesia (<1 ng/mL). The results of our study suggest that previous recommendations for IV CRI doses of hydromorphone (0.03 mg/kg/h) are higher than necessary to achieve human therapeutic targets for hydromorphone. Further work is necessary to investigate whether the human target is also appropriate for dogs.

Constant rate infusions are used to achieve a steady plasma concentration for a drug and might minimize the adverse effects of medications compared to their use with bolus dosing. Intravenous hydromorphone exhibits a rapid clearance and short half-life in dogs, making its administration ideal for CRI. Previously the pharmacokinetics of hydromorphone IV bolus dosing were evaluated (4, 6). This data was used to extrapolate that a CRI of 0.03 mg/kg/h would reach 4 ng/mL steady state in 2 h (6). Preliminary data based on a subcutaneous infusion study postulated the 0.03 mg/kg/h CRI may be higher than necessary to achieve therapeutic levels (8). No previous CRI studies on hydromorphone in dogs exist but studies reporting single IV bolus data were compared to our results. Some parameter estimates from our study are similar to previous IV bolus studies in dogs that utilized either compartmental or noncompartmental analyses and Beagle dogs (4, 6). For example, the volume of distribution at steady state (Vdss) in our study was 4.5 L/kg. Previously reported Vdss values following a bolus of 0.1 mg/kg were 4.2 L/kg (6), 4.5 L/kg (4), and 7.2 L/kg for a bolus of 0.2 mg/kg (4). There were interesting differences in the total body clearance compared to those values previously reported. Our results revealed a clearance of 30.4 mL/min/kg, which is nearly identical to hepatic blood flow in the dog (32 mL/min/kg) (16, 17). Previous studies reported 106 mL/min/kg (6) and 68 mL/min/kg (4), which was far greater than hepatic blood flow and while the authors speculated that extrahepatic metabolism could account for these values, this has never been investigated. Hydromorphone is metabolized by the liver and dogs are well known for their capacity to metabolize opioid drugs (18). Differences in analytical assays, pharmacokinetic methods, and dosing regimens may all account for the variation reported in clearance, and perhaps additional studies could help further characterize the clearance of hydromorphone.

Hydromorphone is a mu opioid agonist that is seven times more potent than morphine. The quality and efficacy of analgesia provided to dogs by hydromorphone compared to fentanyl, butorphanol, oxymorphone have been described (7, 19, 20). However, numerous adverse effects are reported with hydromorphone boluses including nausea, vomiting, defecation, excitation, dysphoria, panting, bradycardia, and respiratory depression (18). In our study, nausea scores were increased during the first 4-h of hydromorphone infusion, which corresponded to plasma concentrations between 9 and 41 ng/mL. Interestingly, the nausea scores returned to nearly normal baseline values quickly, while dogs had higher sedation scores for the duration of the infusion. The sedation scores were consistent with observations of dogs that were subjectively quiet, alert, and responsive. The sedative effect of hydromorphone CRI in systemically sick dogs or when combined with other sedative drugs is not yet known. There is potential for increased risk of adverse effects in these instances (e.g., aspiration risk).

There are several limitations of this study, including the small number of dogs investigated and the uniformity of this cohort of the dogs (Beagle dogs of a similar bodyweight). While this homogeneity is preferred for pharmacokinetic evaluation as it limits variability in data, it also excludes differences that could be seen in other breeds, conformations, or genetic make-up of dogs. While we anticipate the results of this study can be applied to populations of animals experiencing pain, further work is necessary to evaluate the efficacy of hydromorphone CRIs in clinical cases.

## 5 Conclusion

Hydromorphone administered as an IV bolus and CRI for 48 h at a dose of 0.01 mg/kg/h resulted in steady state plasma concentrations above the minimum effective plasma concentration in people and was cleared rapidly upon discontinuation of the infusion. Minimal effects on nausea scores and heart and respiratory rates were observed, while sedation was observed for the duration of the infusion in dogs.

## Data availability statement

The raw data supporting the conclusions of this article will be made available by the authors, without undue reservation.

## Ethics statement

The animal study was approved by North Carolina State University. The study was conducted in accordance with the local legislation and institutional requirements.

## Author contributions

CW: Writing – original draft, Writing – review & editing. AL: Writing – original draft, Writing – review & editing. HK: Writing – original draft, Writing – review & editing. YU: Writing – original draft, Writing – review & editing. KM: Writing – original draft, Writing – review & editing.

## Footnotes

^1^Dexdomitor, Zoetis, Parsippany, NJ.

^2^Jorvet Extended Use IV Catheter, Jorgensen Laboratories LLC, Loveland, CO.

^3^Antisedan, Zoetis, Parsippany, NJ.

^4^BD Insyte, BD Infusion Therapy Systems Inc., Sandy, UT.

^5^Hydromorphone hydrochloride, Pfizer, New York, NY.

^6^BD Vacutainer; Franklin Lakes, NJ.

^7^VWR International Radnor, PA, United States.

^8^Phoenix Winnonlin Version 8.0; Pharsight, NJ, United States.

^9^Graphpad Prism version 7.04, GraphPad Inc.
