# Supplementary material for: Pharmacokinetics of a continuous intravenous infusion of hydromorphone in healthy dogs
Source: Front Vet Sci. 2024 Apr 15;11:1362730. doi: 10.3389/fvets.2024.1362730 (PMC11056520; doi:10.3389/fvets.2024.1362730)
Supplement: Supplementary file 1 [file Table_1.DOCX]

Supplementary Material

**Supplementary Table 1**Seven-point sedation scale validated in dogs by Wagner et al. 2017.

| **1. Spontaneous Posture** | Standing = 0 Tired but standing = 1 Lying but able to rise = 2 Lying but difficult rising = 3 Unable to rise = 4 |
| --- | --- |
| **2. Palpebral Reflex** | Brisk = 0 Slow but with full corneal sweep =1 Slow but with only partial corneal sweep =2 Absent =3 |
| **3. Eye Position** | Central =0 Rotated forwards/downwards but not obscured by third eyelid =1 Rotated forwards/downwards and obscured by third eyelid =2 |
| **4. Jaw and tongue relaxation** | Normal jaw tone, strong gag reflex = 0  Reduced tone, but still moderate gag reflex =1 Much reduced tone, slight gag reflex =2 Loss of jaw tone and no gag reflex = |
| **5. Response to noise (handclap)** | Normal startle reaction (head turn towards noise/ cringe) = 0  Reduced startle reaction (reduced head turn/ minimal cringe) = 1 Minimal startle reaction =2 Absent reaction =3 |
| **6. Resistance when laid into lateral recumbency** | Much struggling, perhaps not allowing this position =0  Some struggling, but allowing this position =1 Minimal struggling/ permissive =2 No struggling = 3 |
| **7. General appearance / attitude** | Excitable =0  Awake and normal =1 Tranquil =2 Stuporous =3 |
