# Supplementary material for: Pharmacokinetics of a continuous intravenous infusion of hydromorphone in healthy dogs
Source: Front Vet Sci. 2024 Apr 15;11:1362730. doi: 10.3389/fvets.2024.1362730 (PMC11056520; doi:10.3389/fvets.2024.1362730)

Supplementary Material

**Supplementary Figure 1A-E** Observed hydromorphone plasma concentrations (ng/mL) [open red circles] versus time (hours) for 6 individual dogs over the duration of the study (0-60 hours). Each dog was administered an intravenous bolus of hydromorphone (0.1 mg/kg) followed by a hydromorphone intravenous constant rate infusion (0.01 mg/kg/hour) for 48-hours. The pharmacokinetic predicted model is indicated by the solid blue line.


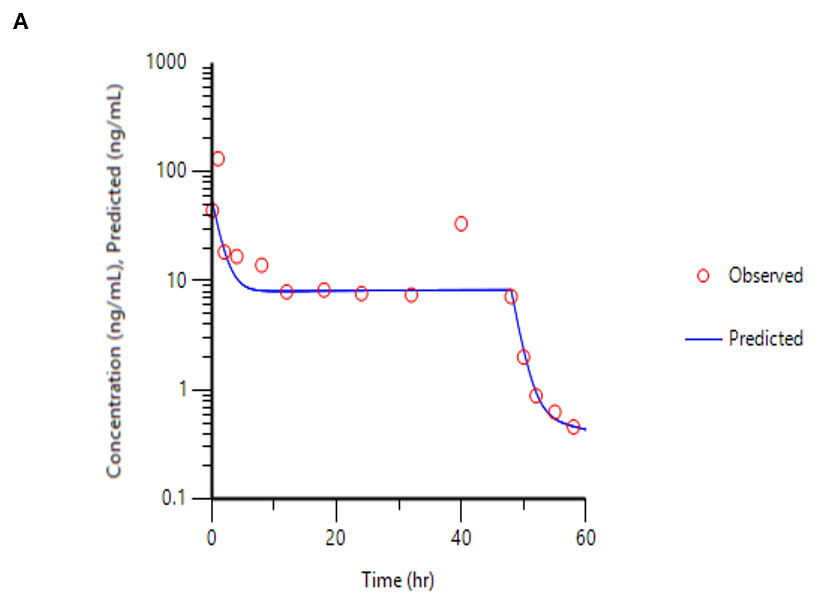

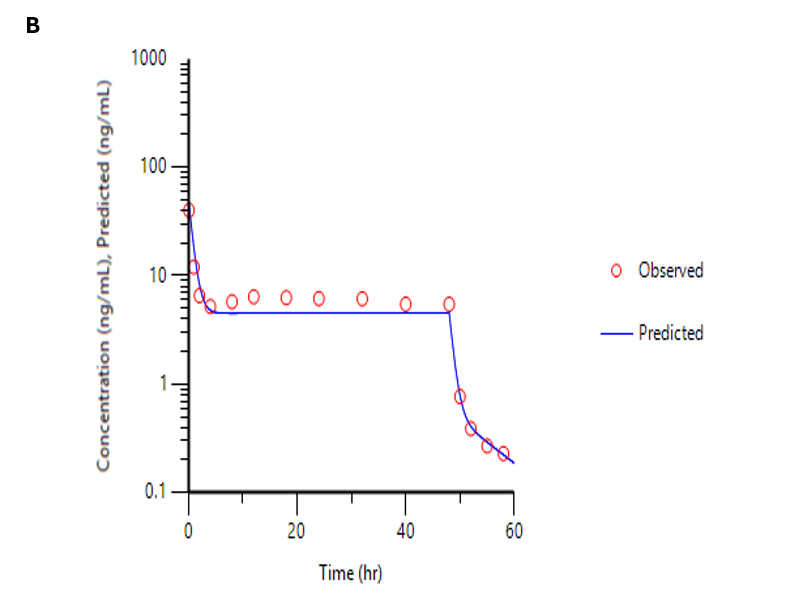


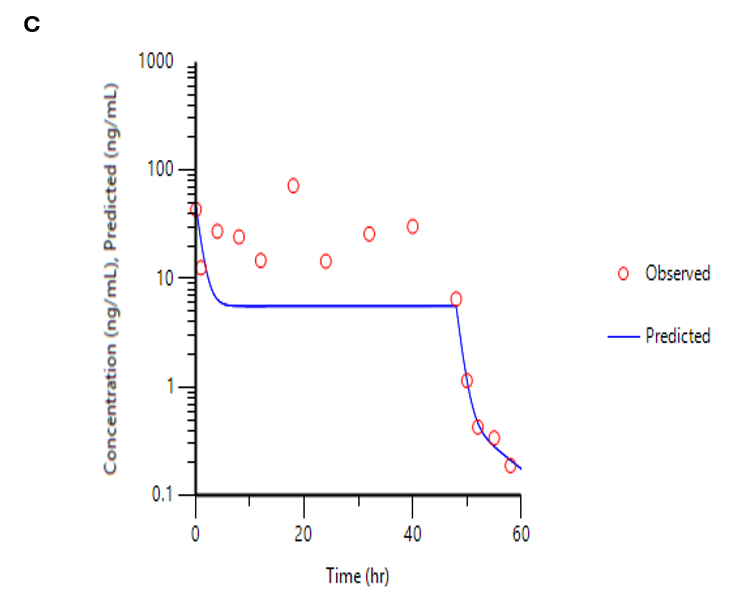

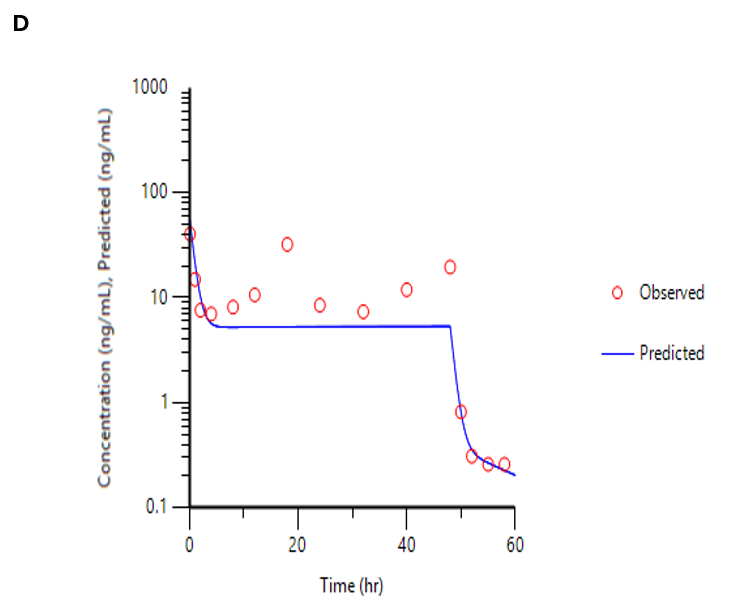


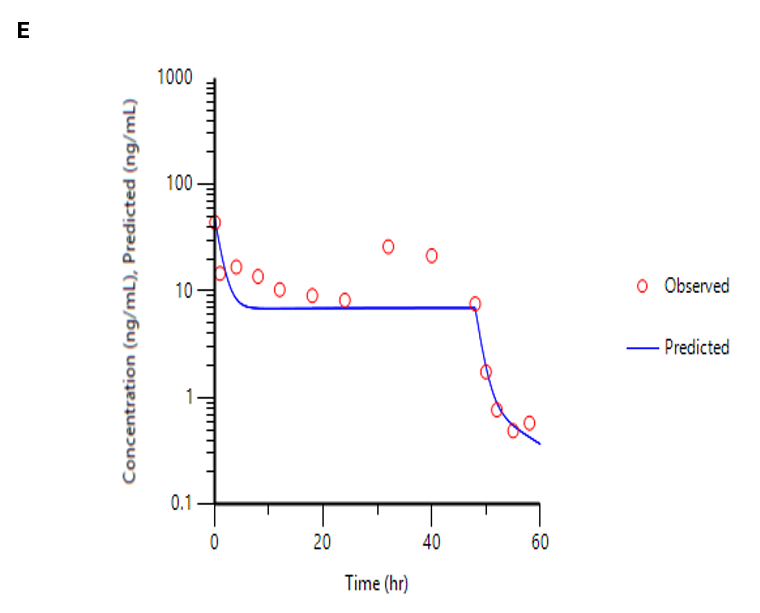

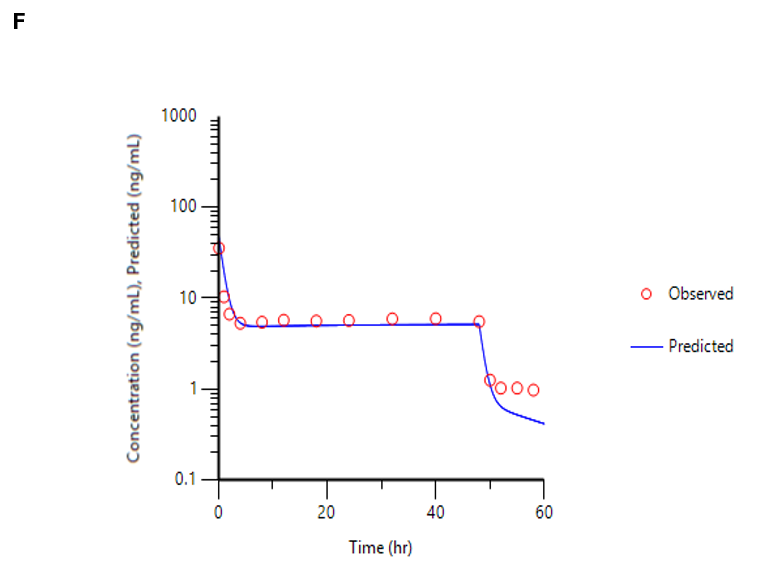

Supplement: Supplementary file 2 [file Table_2.DOCX]
