# Supplementary material for: Pharmacokinetics of a continuous intravenous infusion of hydromorphone in healthy dogs
Source: Front Vet Sci. 2024 Apr 15;11:1362730. doi: 10.3389/fvets.2024.1362730 (PMC11056520; doi:10.3389/fvets.2024.1362730)
Supplement: Supplementary file 3 [file Table_3.DOCX]

Supplementary Material

**Supplementary Figure 2A-E** Weighted residual (WResidual) Y plots versus predicted Y plots for individual dogs over the duration of the study (0-60 hours). Each dog was administered an intravenous bolus of hydromorphone (0.1 mg/kg) followed by a hydromorphone intravenous constant rate infusion (0.01 mg/kg/hour) for 48-hours.

**
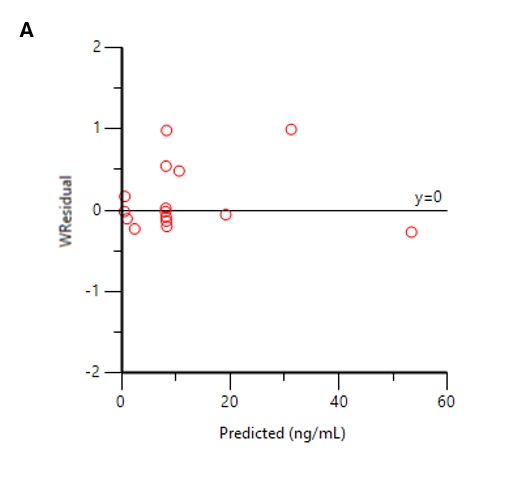

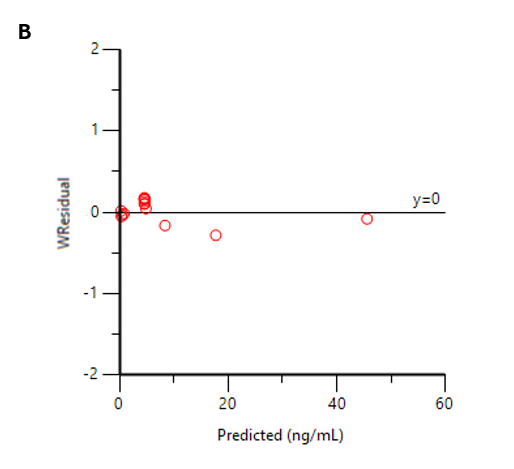
**

**
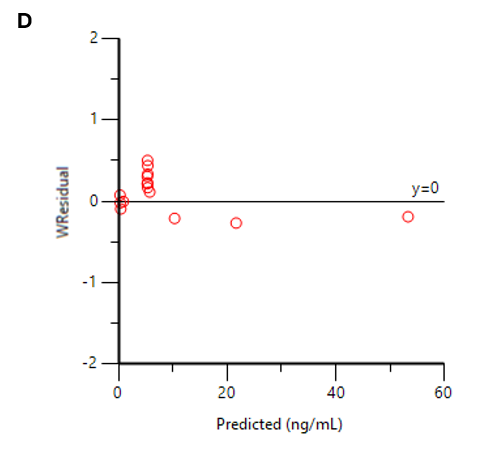

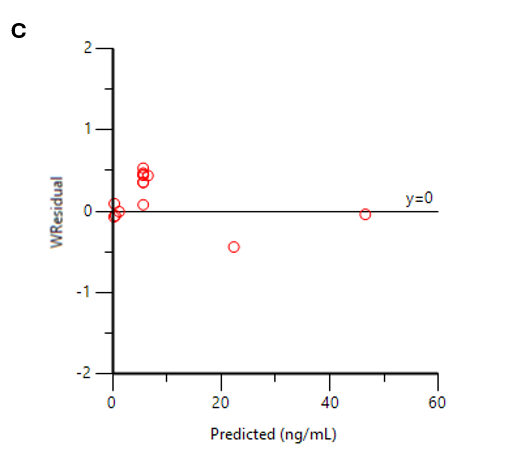
**

**
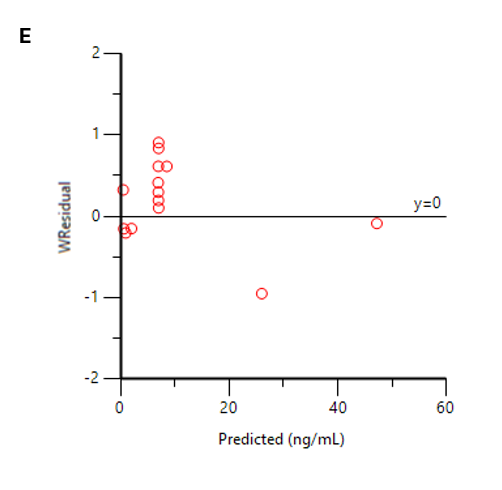

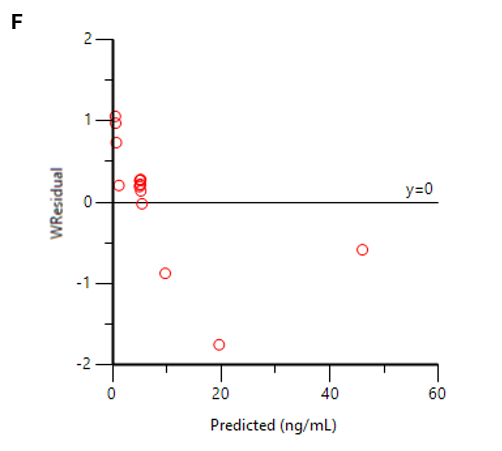
**
